# Quadratus lumborum block type 2 for pedicle groin flap analgesia: a case report

**DOI:** 10.1186/s40981-020-00342-7

**Published:** 2020-05-17

**Authors:** Tatsunori Watanabe, Koji Moriya, Hiroshi Baba

**Affiliations:** 1 Division of Anesthesiology, Uonuma Institute of Community Medicine, Niigata University Medical and Dental Hospital, 4132 Urasa, Minami-Uonuma, Niigata, 949-7302 Japan; 2 Niigata Hand Surgery Foundation, Niigata, Japan; 3 Division of Anesthesiology, Niigata University Graduate School of Medical and Dental Sciences, Niigata, Japan

**Keywords:** Quadratus lumborum block type 2, Pedicle groin flap, Postoperative analgesia

To the Editor,

Quadratus lumborum block type 2 (QLB2) is used to provide lateral and lower abdominal sensory block [1]. Herein, we describe a case involving the use of QLB2 for analgesia of a pedicle groin flap. Patient consent was obtained for publication of this report. The patient was a 51-year-old man (height, 168 cm; weight, 65 kg) who had his left hand crushed in an industrial press machine more than a month prior to the current presentation. Replantation of the middle and ring fingers was performed 1 month prior; however, these fingers became necrotic. Therefore, he was scheduled for a left pedicle flap procedure to address the necrosis. His medical history was unremarkable.

With standard monitoring and no premedication, general anesthesia was induced with propofol (100 mg), remifentanil (0.25 μg/kg/min), and rocuronium (20 mg). After insertion of the #4 laryngeal mask airway, general anesthesia was maintained with sevoflurane (1.5%) and remifentanil (0.1 μg/kg/min) (FiO_2_: 0.4). A left supraclavicular brachial plexus block followed by QLB2 were performed under ultrasound guidance using 30 mL of 0.25% ropivacaine, respectively (total, 150 mg ropivacaine). For the QLB2 procedure, a linear transducer was placed in the axial plane in the midaxillary line and moved posteriorly until the quadratus lumborum was confirmed. The target was posterior to the quadratus lumborum, and the needle tip was advanced near the lumbar interfascial triangle. Local injections were administered between the area posterior to the quadratus lumborum and outside the middle layer of the thoracolumbar fascia.

The surgical procedure included debridement of the hand, followed by the creation of a pedicle groin flap at the left inguinal region (Fig. 1). No increases in blood pressure or heart rate were noted during surgery. At the end of the surgery, infusion of anesthetics was discontinued, sugammadex (200 mg) was administered, and the laryngeal mask airway was removed. The patient reported only pre-existing shoulder pain but no wound pain (numerical rating scale 0/10) even though no additional analgesics were administered postoperatively. On postoperative day 1, he received loxoprofen (60 mg) three times. He subsequently reported a wound pain (on a numerical rating scale) of 1/10 at rest and 3/10 while in motion. Three weeks later, the second surgery to divide the pedicle was performed under local infiltration anesthesia.

**Fig. 1:**
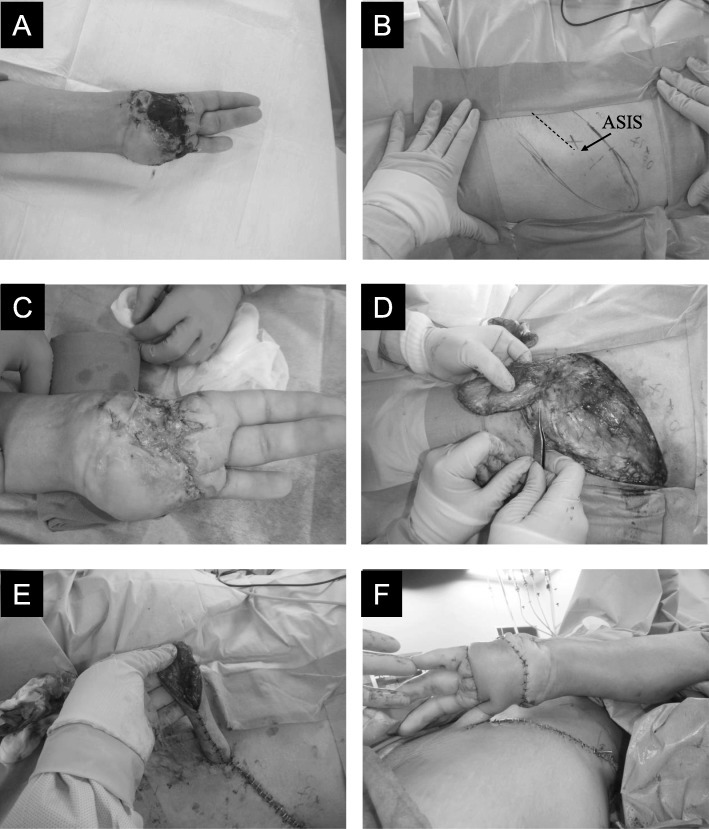
**a** Injured left hand with necrosis. Both the thumb and the index finger were missing. **b** The donor site of the pedicle groin flap in the left inguinal region. The dotted line between the anterior superior iliac spine (ASIS) and the femoral artery 2–2.5 cm below the inguinal ligament is drawn (axial line of the flap). **c** Debridement of the left hand. **d** Construction of the flap. **e** Tubed pedicle. **f** The flap sutured in position

QLB2 and brachial plexus nerve block provided good postoperative analgesia in the present case, and there was evidently no need for opioids. QLB2 has been previously reported for analgesia in the T10–T12 or L1 in the cranial-caudal direction and in the area around the posterior axillary line on the dorsal side and over the linea semilunaris on the ventral side [1]. Although it is unknown whether QLB2 contributed to anesthesia during the creation of the pedicle groin flap, it was demonstrably effective for postoperative analgesia at the site.

Notably, a single block has been previously reported to provide sufficient effects during soft tissue surgery [2]. Similarly, a single dose provided sufficient effects in the current case.

## Data Availability

Please contact author for data requests.

## Abbreviations

QLB2: Quadratus lumborum block type 2
ASIS: Anterior superior iliac spine
